# Child- and Parent-Related Correlates of Total and Prolonged Sedentary Time in 5- to 6-Year-Old Children

**DOI:** 10.3390/ijerph15091817

**Published:** 2018-08-22

**Authors:** Evi van Ekris, Emma Solomon-Moore, Mai J. M. Chinapaw, Russell Jago, Teatske M. Altenburg

**Affiliations:** 1 Department of Public and Occupational Health, Amsterdam UMC, Vrije Universiteit Amsterdam, Amsterdam Public Health Research Institute, 1081 BT Amsterdam, The Netherlands; m.chinapaw@vumc.nl (M.J.M.C.); t.altenburg@vumc.nl (T.M.A.); 2 Department for Health, University of Bath, Claverton Down, Bath BA2 7AY, UK; E.Solomon-Moore@bath.ac.uk; 3 Centre for Exercise, Nutrition & Health Sciences, School for Policy Studies, University of Bristol, Bristol BS8 1UQ, UK; russ.jago@bris.ac.uk

**Keywords:** primary school, children, parents, accelerometry, objective monitoring, sedentary time, cross-sectional

## Abstract

The primary aim was to examine child- and parent-related correlates of accelerometer-assessed overall total and prolonged (i.e., accumulated in bouts of ≥10 consecutive minutes) sedentary time (SED) in 5- to 6-year-old children. Second, child- and parent-related correlates of total and prolonged SED during weekend days and the after school period were examined, as associations with parent-related correlates may be stronger during these periods. SED and moderate-to-vigorous-intensity physical activity (MVPA) were assessed by ActiGraph accelerometers in children (*n* = 836) and one of their parents/carers. Parents completed a questionnaire examining potential parent-related correlates. Multilevel models examined associations between potential correlates and children’s total and prolonged SED. Children’s MVPA was the only correlate that was consistently negatively associated with both total and prolonged SED across the different time periods (overall, after school, and weekend days). Higher total SED in parents was associated with higher overall total SED and weekend total SED in children. Higher body mass index *z*-scores of children were associated with lower overall total and prolonged SED. Girls had lower prolonged SED after school than boys. Older children had lower total SED during the weekend. In conclusion, few potential correlates were associated with young children’s total or prolonged SED and most associations differed by time period.

## 1. Introduction

Sedentary behavior is defined as any waking behavior characterized by an energy expenditure less than or equal to 1.5 metabolic equivalents while in a sitting, reclining, or lying posture [1]. Sedentary time (SED) is the time spent engaging in sedentary behaviors for any duration or in any context [1]. Current UK guidelines state that children 5–18 years old should minimize their amount of SED for extended periods [2]. Nevertheless, estimates suggest that European children spend up to 9 h per day being sedentary [3]. SED in adults has been related to all-cause mortality, cardiovascular disease mortality, and type 2 diabetes [4]. Some evidence indicates that children’s SED, in particular TV-viewing time, is related to an increased risk for being overweight and having decreased fitness [5], but evidence for detrimental health effects of SED in children is generally inconclusive [5]. One possible explanation may be that children have not yet been exposed to sedentary time long enough to develop ill health [6]. However, sedentary habits established in childhood persist with moderate tracking over time, and may ultimately track into adulthood [7]. Therefore, childhood is a critical period to limit SED, highlighting the need for effective evidence-based interventions at an early age.

Current evidence indicates that the majority of interventions to reduce SED in children have not been effective [8]. This may be explained by lack of knowledge regarding the most important correlates and determinants of children’s SED [8]. Parent-related correlates may be important to young children’s SED, as young children have little autonomy and parents play an essential role in their health behaviors [9]. It is currently unknown which parent-related characteristics are important in influencing young children’s SED, as studies are heterogeneous in investigated characteristics [10,11,12]. In their systematic review, Xu et al. [12] summarized the evidence for associations of (1) parenting practices, (2) parents’ role-modeling, (3) parents’ perceptions of children’s physical activity (PA) and screen-viewing behaviors, (4) parental self-efficacy, and (5) general parenting style with screen viewing in young children (aged ≤ 6 years). Their results suggest that parental role-modeling may be important for young children’s SED, as evidence was found for an association between parents’ screen time and young children’s screen time [12]. Moreover, Xu et al. [12] found evidence for an association between higher parental self-efficacy for reducing screen time and less screen time in young children. However, this evidence is based on screen time rather than total SED. Besides parent-related correlates, child-related correlates, like body mass index (BMI) and moderate-to-vigorous-intensity physical activity (MVPA), may be associated with total SED in 9- to 11-year-old children [13]. However, associations with objectively measured total SED are examined only in few studies in young children [10].

As it is currently unclear which child- and parent-related correlates are most important for young children’s total SED, more research is needed to inform future interventions aimed at reducing young children’s SED. Importantly, previous studies on parent- and child-related correlates of young children’s SED focused predominantly on subjectively measured screen and/or TV-viewing time rather than objectively measured total SED [10]. Verloigne et al. [14] found that screen and TV-viewing time do not make a good proxy for total SED in 10- to 12-year-old children. Moreover, the study of Le Blanc et al. [15] among 10-year-old children indicates that correlates for total SED may be different to correlates for screen time. Correlates of TV-viewing and screen time may also be different to correlates for total SED in young children, and thus knowledge about correlates of objectively measured total SED among young children is needed. Moreover, correlates of prolonged, uninterrupted SED in young children have not been studied before. Studying correlates of prolonged SED may be important, because a recent acute study in young adults (18 to 24 years old) [16] and epidemiological studies in children (8 to 11 years old) [17,18] suggest that the extent to which total SED is accumulated in bouts of prolonged uninterrupted SED may be important for health outcomes.

The present study aimed to identify child- and parent-related correlates of objectively measured total and prolonged SED in 5- to 6-year-old children. A secondary aim was to examine child- and parent-related correlates of total and prolonged SED during weekend days and the after school period, as associations with parent-related correlates may be stronger during these periods.

## 2. Materials and Methods 

The present study used data from the B-Proact1v study, which aimed to examine factors associated with young children’s and parents’ MVPA and SED. A detailed study description can be found elsewhere [19]. Briefly, 250 primary schools located within the greater Bristol area (UK) were invited to participate in the study, of which 57 schools agreed to participate. All Year 1 pupils, or Year 1/2 for combined classes, were eligible to take part, with 1456 children from a potential 2600 pupils (56%) participating (age range 4–7 years). One parent/carer was required to participate with each child. Data were collected between January 2012 and May 2013. The B-Proact1v study was approved by the School for Policy Studies ethics committee at the University of Bristol. Written parental consent was obtained for all participants.

### 2.1. Accelerometer Measurements

SED and MVPA of children and parents were objectively assessed using ActiGraph wGT3X (Pensacola, FL, USA) accelerometers. Children and parents were asked to wear an accelerometer on their waist during all waking hours for 5 consecutive days, including 2 weekend days. Raw accelerometer data were processed in MATLAB version R2009a (Natick, MA, USA) in 60 s epochs, using a customized software program [20]. Primary analyses were aimed at average daily total and prolonged SED of a representative week (i.e., overall total SED and overall prolonged SED). To be included in the primary analyses, children and parents needed to provide accelerometer data for at least 4 valid days, including at least 1 weekend day. A day was defined as valid when the accelerometer was worn for at least 8 h. Non–wear time was defined as 60 min or more of consecutive zeros [20,21]. Total SED was determined using a cut-point of <100 counts per minute (CPM) for both children and parents [21,22]. Prolonged SED was defined as total time accumulated in sedentary bouts of at least 10 consecutive minutes <100 CPM, allowing no tolerance time within sedentary bouts [23]. MVPA was calculated by applying the Evenson cut-point for children (≥2296 CPM) [22] and the Troiano cut-point for parents (≥2020 CPM) [24]. Children’s total and prolonged SED were included as outcomes, while children’s MVPA, parents’ MVPA, and parents’ total and prolonged SED were included as potential correlates. Only children with valid data for the primary analysis were included in the secondary analysis of weekend and after school SED. Two valid weekend days were required for the weekend analysis. For the after school time analysis, children needed to have at least 3 hours of data after 15:30 (until the accelerometer was taken off) on at least 2 weekdays.

### 2.2. Potential Correlates

Potential correlates of children’s total and prolonged SED included various child- and parent-related characteristics. Child characteristics included MVPA, BMI *z*-score, age, and gender. Trained researchers measured children’s weight and height. Weight was measured to the nearest 0.1 kg using a calibrated Seca 899 digital scale (HAB International, Northampton, UK). Height was measured to the nearest 0.1 cm with a portable Seca Leicester stadiometer (HAB International, Northampton, UK). BMI was calculated (weight (kg)/height (m^2^)) and expressed as standardized *z*-score based on gender and age using the UK child growth reference curves [25]. Children were categorized as normal weight, overweight, or obese according to the World Health Organisation cutoffs [26]. Children’s ages were calculated from parent-reported dates of birth. Parent-related characteristics were subdivided into parents’ SED and parenting characteristics. Table A1 provides an overview of the questions on parenting characteristics. Parenting characteristics included: (1) efficacy in influencing children’s screen viewing, assessed using a modified version of Bandura’s self-efficacy scale [27] (mean score over 3 items); (2) restricting child access to screen activities (mean score over 3 items) [28]; and (3) attitudes toward screen viewing (mean score over 8 items) [29]. The internal consistency of the items was calculated using Cronbach’s alpha, and was acceptable (i.e., α > 0.7) for all constructs (Table A1). 

### 2.3. Demographics

Parent demographics were used for descriptive purposes and included age, gender, ethnicity, BMI, and an index of multiple deprivation (IMD) score. Parent BMI was calculated from self-reported weight and height, and parents were categorized as normal weight (<25 kg/m^2^), overweight (25.0–29.9), or obese (≥30.0). The IMD score was assigned using the household postcode and calculated using the 2010 English Indices of Deprivation, with higher scores indicating greater deprivation [30]. IMD is a composite measure of relative deprivation at the lower area level and includes 7 domains: income, employment, health, education, crime, housing and service, and the living environment. 

### 2.4. Statistical Analyses

**Figure 1:**
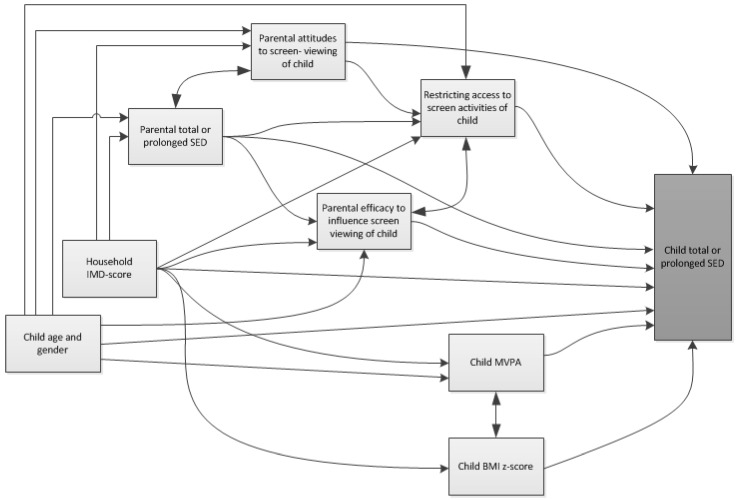
Potential correlates of children’s total and prolonged sedentary time (SED) and their expected associations. IMD, index of multiple derivation; MVPA, moderate-to-vigorous-intensity physical activity; BMI, body mass index.

Means and proportions were used to examine the distributions of potential correlates and outcomes. Questionnaire data measured with Likert scales were treated as continuous variables, as there was no indication that assumptions of normality and linearity were violated. Multicollinearity among potential correlates was tested using Pearson’s correlations. Parents’ total SED was included in the analysis of children’s total SED, and parents’ prolonged SED was included in the analysis of children’s prolonged SED. Figure 1 visualizes how the potential correlates were expected to be associated with children’s SED. It was not the purpose of the present study to statistically analyze all paths drawn in Figure 1 (e.g., using directed acyclic graph analyses [31]), because this would require longitudinal data. Instead we developed Figure 1, based on the literature and the authors’ knowledge, with the purpose of selecting the most appropriate potential correlates out of all measured variables. We considered a measured variable to be a potential correlate if it was expected to demonstrate a direct association with children’s SED. Parent BMI (not displayed in Figure 1) was not included as a potential correlate, as it was expected to indirectly influence children’s SED via other measured variables like child BMI and parent SED. IMD score was considered a confounder for most associations rather than a correlate itself (it was expected to influence children’s SED through other measured or unmeasured variables). Additionally, Figure 1 was used to select the most appropriate set of confounders for each association (i.e., variables expected to be associated with both a potential correlate and children’s SED). The association of each potential correlate with children’s SED was adjusted for a specific set of confounders based on Figure 1 (see Table 1). To determine correlates of children’s total and prolonged SED, multilevel linear regression analyses with school as a random variable were applied. Besides the specific set of confounders for each potential correlate, associations were additionally adjusted for children’s accelerometer wear time and, where relevant, parents’ accelerometer wear time (accelerometer wear time is not included in Figure 1). Analyses were conducted in SPSS 20.0 (SPSS, Inc., Chicago, IL, USA).

## 3. Results

Table 2 presents the characteristics of children and their parents/carers. In total, 863 children (52% boys, mean age 6.0 ± 0.4 years) provided valid accelerometer data and were included in the primary analysis. Accelerometer data during weekend days and after school time were available for 605 and 797 children, respectively. Data on potential correlates were available for 738–859 children, depending on the specific potential correlate.

The majority of the participating parents were mothers (75%) and declared themselves as white British (90%). The average IMD score was 14, indicating that our sample was slightly less deprived than the average in England (the IMD score of an average area was 17) [29]. Children spent on average 289 min sedentary each day, of which 121 min was prolonged SED. Parents of children who did not provide data in the subsample for weekend analysis (*n* = 257 children had no valid data) had lower daily average accelerometer wear time and total and prolonged SED. Other main characteristics were similar between the samples (Table A2).

Table 3 presents the associations of child- and parent-related correlates with children’s total and prolonged SED. Higher child MVPA was associated with lower total and prolonged SED across all time periods. Higher BMI-z was associated with lower overall total (β = −5.16 (95% confidence interval (CI): −8.26; −2.06)) and prolonged SED (−3.18 (−6.23; −0.13)), but not with SED during weekends or after school. Associations of child gender and age with SED were time period–specific: girls had lower prolonged SED after school than boys (−6.81 (−11.03; −2.59)), and older children had lower total SED during the weekend than younger children (−13.25 (−25.91; −0.58)). Parents’ total SED was positively associated with children’s overall total SED (0.06 (0.02; 0.11)) and weekend total SED (0.11 (0.04; 0.17)). Parents’ prolonged SED was not associated with children’s prolonged SED. Parenting characteristics were not associated with children’s total or prolonged SED.

## 4. Discussion

The primary aim of the current study was to examine child- and parent-related correlates of overall total and prolonged SED in 5- to 6-year-old children. Secondarily, we examined child- and parent-related correlates of total and prolonged SED during weekend days and after school time. This study was the first to examine correlates of prolonged SED in young children. Only a few potential correlates were associated with children’s total or prolonged SED across at least one of the time periods (overall, weekend, and after school). Higher child MVPA was the only correlate that was consistently associated with lower total and prolonged SED across all time periods in young children. Associations of child BMI *z*-score, parents’ total SED, child age, and child gender with children’s total or prolonged SED were time period–specific.

We found that none of the parenting-related potential correlates were associated with total or prolonged SED. This lack of association may be explained by limited variability in the responses to the parenting-related characteristics, with parents generally reporting favorable responses, potentially due to social-desirability bias [32]. Secondly, the parenting-related characteristics were aimed at screen time instead of total SED. It is possible that parents actively restrict screen time but not nonscreen sedentary activities (e.g., playing with Lego, doing crafts, reading) [33]. In line with our findings, systematic reviews generally indicate inconclusive evidence for associations between parenting factors and children’s SED, although conclusions of these systematic reviews are mainly based on studies examining TV viewing and screen time [11,12]. Some evidence suggests that parents’ screen time is related to children’s screen time [12]. This is in line with our finding that children’s total SED overall and during weekends was lower when their parents’ total SED was lower. In contrast to parents’ total SED, parents’ prolonged SED was not associated with children’s prolonged SED.

Regarding the child-related characteristics, we found that children’s overall total and prolonged SED were lower when their BMI *z*-scores were higher. Previous studies have shown that young children with higher BMI have a higher total SED [34,35] or have reported null associations [36,37]. It could be that parents of children with higher BMI *z*-scores in this sample were already actively trying to reduce their children’s SED. Another explanation may be that the children in our sample had a healthier weight than the average. In our sample, only 3% of the children were classified as obese, whereas on average about 10% of children 5 years of age are obese in the UK [38]. Higher child MVPA was the only correlate that was consistently associated with lower total and prolonged SED across the different time periods. Betas ranged between −0.63 and −1.38, meaning that each additional minute of MVPA resulted in 0.63 to 1.38 min less SED. This supports the displacement hypothesis, which states that increased SED may hinder MVPA and vice versa [39]. However, Pearson et al. [40] concluded in their meta-analysis only a small negative association between SED and MVPA, although slightly stronger associations were reported in studies measuring SED objectively. Child age and gender are not modifiable but were included as potential correlates, because we expected that they were directly associated with children’s SED. Child age and gender were not important correlates in this study and observed associations with SED were time period–specific. The age range was 4 to 7 years, which may be too narrow to detect substantial behavioral differences. 

Most of the identified correlates in this study were time period–specific, and we found differences in correlates of total and prolonged SED. This is in line with a study on determinants of SED by Janssen et al. [41], although that study followed children from 9 to 12 years of age and used another indicator of sedentary patterns, sedentary fragmentation, or the extent to which SED is prolonged versus interrupted. We identified only a few modifiable correlates, often with weak associations. Systematic reviews indicate, in general, inconclusive evidence on correlates and determinants of young children’s total SED due to inconsistent findings, null findings, or too few studies [10,42]. Therefore, potential correlates that have been investigated thus far may not be the most important correlates of children’s SED. Although the B-Proact1v study was specifically designed to examine correlates of SED, most previous studies were not [43]. In order to provide new insights into child- and parent-related correlates of importance, qualitative studies exploring children’s and parents’ motives for children to engage in SED are required [44]. Potential correlates identified in these studies should subsequently be tested in cohort studies for strength of association. 

A key strength of this study is the objectively assessed SED in both children and parents.

Most previous studies on correlates of young children’s SED focused on subjectively measured TV viewing and screen time. This study was the first to examine correlates of prolonged SED in young children. Another strength is that we explored potential correlates of children’s SED based on a hypothetical model (Figure 1), ensuring that each association was adjusted for the most appropriate set of confounders. A limitation is the cross-sectional design, which makes inferences about causality impossible. Next, the correlates based on parent reports may be affected by social-desirability bias, and some correlates were aimed at screen time. Another limitation is that parent-reported correlates were obtained from only one parent. Finally, although our sample was relatively large, there was little ethnic diversity and participants were slightly less deprived than the average population. Our sample is therefore not representative of the whole population and caution should be taken when generalizing our findings.

## 5. Conclusions

Only a few of the examined potential child- and parent-related correlates were associated with young children’s total or prolonged SED, and most associations differed by time period. Higher child MVPA was the only correlate that was consistently associated with lower total and prolonged SED across all time periods. Higher MVPA was associated with a comparable lower SED, indicating some displacement. Future qualitative studies exploring children’s and parents’ motives for children to engage in SED are required to provide insights into potential relevant correlates.

## Figures and Tables

**Table 1:** Adjustment of associations between child- and parent-related potential correlates with children’s total and prolonged sedentary time *.

Potential Correlates	Included Confounders in Association with Children’s Total and Prolonged SED *
Child BMI *z*-score	Child wear time, MVPA, IMD score, gender
Child overall MVPA ^a^	Child wear time, IMD score, gender, age, BMI *z*-score
Child age	Child wear time
Child gender (male is reference)	Child wear time
Parents’ total and prolonged SED	Child wear time, parent wear time, IMD score, child gender, child age
Parental efficacy in influencing child’s screen viewing	Child wear time, parent wear time, IMD score, child gender, child age, parental total or prolonged SED ^a^ restricting access to child’s screen activities
Restricting access to child’s screen activities	Child wear time, parent wear time, IMD score, child gender, child age, parental total or prolonged SED ^a^ parental efficacy in influencing child’s screen viewing, parental attitudes on child’s screen viewing
Parental attitudes on child’s screen viewing	Child wear time, parent wear time, IMD score, child gender, child age, total or prolonged SED ^a^ restricting access to child’s screen activities

* Set of confounders used for each association was selected based on Figure 1. BMI, body mass index; MVPA, moderate-to-vigorous-intensity physical activity; SED, sedentary time; IMD, indices of multiple deprivation. ^a^ Parents’ total sedentary time was included in the analysis of children’s total sedentary time and parents’ prolonged sedentary time was included in the analysis of children’s prolonged sedentary time.

**Table 2:** Characteristics of 5- to 6-year-old children and their parents/caregivers.

Characteristics	Mean ± SD	*n*
*Child characteristics*		
Age (years)	6.0 ± 0.4	756
Gender (% boys)	52%	859
BMI *z*-score	0.2 ± 0.9	859
Weight status		859
Normal weight	82%	
Overweight	15%	
Obese	3%	
*Child SED and MVPA*		
Overall		863
Total SED (min/day)	289 ± 66	
Prolonged SED (min/day)	121 ± 55	
MVPA (min/day)	52 ± 21	
Accelerometer wear time (min/day)	706 ± 74	
Weekend		605
Weekend total SED (min/day)	278 ± 86	
Weekend prolonged SED (min/day)	121 ± 74	
Weekend MVPA (min/day)	50 ± 27	
Weekend accelerometer wear time (min/day)	682 ± 97	
After school		797
After school total SED (min/day)	119 ± 38	
After school prolonged SED (min/day)	60 ± 34	
After school MVPA (min/day)	20 ± 12	
After school accelerometer wear time (min/day)	269 ± 47	
*Parent characteristics*		
Relationship of parent to child (%)		
Mother	75%	782
Father	25%	
Other carer	0.40%	
Parent age (years)	38 ± 6	
Parent ethnicity (%)		782
White British	90%	
White other	5%	
Other	6%	
Parent BMI (kg/m^2^)	25 ± 4	755
Parent weight status (%)		755
Normal weight	56%	
Overweight	30%	
Obese	14%	
Multiple deprivation (IMD) score	14 ± 12	824
*Parents’ overall SED and MVPA*		
Parents’ total SED (min/day)	475 ± 101	738
Parents’ prolonged SED (min/day)	271 ± 105	738
Parents’ MVPA (min/day)	35 ± 21	738
Parents’ accelerometer wear-time (min/day)	837 ± 95	738
*Parenting characteristics*		
Parental efficacy in influencing child’s screen viewing (range: 1–5) ^a^	4.6 ± 0.6	777
Restricting access to child’s screen activities (range: 1–4) ^b^	3.4 ± 0.6	771
Parental attitudes on child’s screen viewing (range: 1–5) ^c^	3.8 ± 0.7	739

Data are presented as mean ± SD, unless otherwise stated. Note that the percentages do not always add up to 100% because of rounding. BMI, body mass index; MVPA, moderate-to-vigorous-intensity physical activity; SED, sedentary time; IMD, indices of multiple deprivation; *n*, number of participants with available data on correlate. ^a^ 1 = nothing to 5 = a great deal; ^b^ 1 = strongly disagree to 4 = strongly agree; ^c^ 1 = beneficial to 5 = harmful.

**Table 3:** Associations of child- and parent-related correlates with children’s total and prolonged sedentary time.

Correlates	Overall			Weekend			After School		
	β (95% CI)	*p*-Value	*n*	β (95% CI)	*p*-Value	*n*	β (95% CI)	*p*-Value	*n*
Total sedentary time									
Child BMI *z*-score	−5.16 (−8.26; −2.06)	<0.001	822	−3.14 (−8.11; 1.83)	0.215	583	−0.40 (−2.35; 1.55)	0.685	759
Child overall MVPA ^a^	−1.12 (−1.26; −0.97)	<0.001	747	−0.97 (−1.14; −0.81)	<0.001	540	−1.38 (−1.53; −1.23)	<0.001	687
Child age	−2.18 (−10.04; 5.68)	0.586	755	−13.25 (−25.91; −0.58)	0.040	545	−2.42 (−7.86; 3.01)	0.381	694
Child gender (male is reference)	4.90 (−1.31; 11.11)	0.122	858	3.28 (−6.14; 12.71)	0.494	604	−3.48 (−7.53; 0.56)	0.091	793
Parents’ total SED	0.06 (0.02; 0.11)	0.009	655	0.11 (0.04; 0.17)	0.002	482	0.02 (−0.01; 0.05)	0.169	607
Parental efficacy in influencing child’s screen viewing	−0.59 (−7.60; 6.42)	0.869	646	−0.18 (−10.43; 10.07)	0.973	476	1.93 (−2.59; 6.45)	0.402	600
Restricting access to child’s screen activities	2.88 (−3.46; 9.23)	0.372	623	−2.25 (−11.83; 7.33)	0.645	462	4.10 (−0.10; 8.29)	0.056	579
Parental attitudes on child’s screen viewing	−0.09 (−5.45; 5.27)	0.974	624	1.87 (−6.02; 9.75)	0.642	463	0.61 (−2.92; 4.14)	0.734	580
Prolonged sedentary time									
Child BMI *z*-score	−3.18 (−6.23; −0.13)	0.041	822	−1.26 (−6.43; 3.91)	0.633	583	0.55 (−1.69; 2.80)	0.733	759
Child overall MVPA ^a^	−0.76 (−0.90; −0.62)	<0.001	747	−0.63 (−0.80; −0.45)	<0.001	540	−0.99 (−1.17; −0.81)	<0.001	687
Child age	−3.71 (−10.96; 3.53)	0.315	755	−6.81 (−19.26; 5.64)	0.283	545	−4.72 (−10.07; 0.64)	0.084	694
Child gender (boy is reference)	−2.76 (−8.49; 2.98)	0.346	858	−4.26 (−13.45; 4.93)	0.363	604	−6.81 (−11.03; −2.59)	0.002	793
Parents’ prolonged SED	0.02 (−0.01; 0.06)	0.195	655	0.04 (−0.02; 0.09)	0.173	482	0.01 (−0.02; 0.04)	0.554	607
Parental efficacy in influencing child’s screen viewing	−0.66 (−7.13; 5.81)	0.840	646	2.54 (−7.74; 12.81)	0.628	476	2.44 (−2.37; 7.24)	0.319	600
Restricting access to child’s screen activities	1.62 (−4.29; 7.53)	0.590	623	−0.06 (−9.62; 9.49)	0.989	462	2.79 (−1.68; 7.25)	0.220	579
Parental attitudes to child’s screen viewing	−0.41 (−5.41; 4.59)	0.872	624	0.94 (−6.95; 8.83)	0.815	463	0.91 (−2.84; 4.67)	0.633	580

Multilevel analyses with school as a random variable, associations are adjusted for different sets of confounders (see Figure 1 and Table 1). ^a^ For primary analysis of overall SED, overall MVPA was used. For secondary weekend analysis, weekend MVPA was used. For secondary after school analysis, after school MVPA was used. BMI, body mass index; CI, confidence interval; MVPA, moderate-to-vigorous-intensity physical activity; SED, sedentary time.

## Appendix A

**Table A1:** Descriptions of questionnaire items on parenting characteristics.

Potential Correlate	Questionnaire Items	Measurement Scale	Internal Consistency ^1^
Parental efficacy in influencing child’s screen viewing	1. How much can you do to control the time your child spends screen viewing? (e.g., watching TV, DVDs, playing video games) 2. How much can you do to help your child have alternatives to screen viewing? 3. How much could you do to reduce the time your child spends screen viewing?	5-point Likert (1 = nothing to 5 = a great deal)	α = 0.88
Restricting access to child’s screen activities	1. I limit how long my child plays video games (including PlayStation, Xbox, and handheld game consoles). 2. I limit how long my child can watch TV and DVDs each day (including educational and noneducational programs). 3. I limit how long my child can use the computer for things other than homework (such as playing computer games and surfing the Internet).	4-point Likert (1 = strongly disagree to 4 = strongly agree)	α = 0.89
Parental attitudes on screen viewing	Children spending several hours per day watching television or playing video games is: 1. Beneficial—Harmful 2. Healthy—Unhealthy 3. Useful—Of no use 4. Of no concern—Of concern Children spending several hours per day during leisure time using the computer or surfing the Internet is: 5. Beneficial—Harmful 6. Healthy—Unhealthy 7. Useful—Of no use 8. Of no concern—Of concern	5-point Likert scale (1 = beneficial to 5 = harmful healthy—unhealthy useful—of no use of no concern—of concern)	α = 0.89

^1^ Internal consistency of the items was calculated using Cronbach’s alpha (α).

**Table A2:** Main child- and family-related characteristics of children included in the total sample and children who did not provide data in the subsample for weekend analysis.

Characteristics	Total Sample (*n* = 863)	Participants Who Did Not Provide Data in the Analysis for Weekend Days (*n* = 257)
Child characteristics		
Age (years)	6.0 ± 0.4	6.0 ± 0.5
Gender (% boys)	52%	52%
BMI *z*-score	0.2 ± 0.9	0.3 ± 1.0
Weight status		
Normal weight	82%	79%
Overweight	15%	15%
Obese	3%	5%
Children’s overall SED and MVPA		
Total SED (min/day)	289 ± 66	289 ± 71
Prolonged SED (min/day)	121 ± 55	123 ± 57
MVPA (min/day)	52 ± 21	51 ± 21
Family-related characteristics		
Parent age (years)	38 ± 6	37 ± 6
Parent BMI (kg/m^2^)	25 ± 4	26 ± 5
Multiple deprivation (IMD) score	14 ± 12	16 ± 13
Parents’ overall SED and PA		
SED (min/day)	475 ± 101	460 ± 95
Prolonged SED (min/day)	271 ± 105	260 ± 95
MVPA (min/day)	35 ± 21	36 ± 21
Accelerometer wear time (min/day)	837 ± 95	818 ± 97

Mean ± SD. MVPA, moderate-to-vigorous-intensity physical activity; SED, sedentary time.
